# Prognostic value and predictive model construction for patients undergoing laparoscopic radical prostatectomy based on the preoperative NPL-IRS score and prognostic nutritional index

**DOI:** 10.3389/fonc.2025.1603993

**Published:** 2025-08-20

**Authors:** Hao Wang, Pu-shen Yang, Yi-rui Wei, Da-wei Xie, Si-qi Wang, Wei-feng He, Wei Wang, Jian-wen Wang

**Affiliations:** Department of Urology, Beijing Chaoyang Hospital, Capital Medical University, Beijing, China

**Keywords:** prostate cancer, laparoscopic radical prostatectomy, NPL-IRS score, prognostic nutritional index, prognosis

## Abstract

**Objective:**

To explore the prognostic value of preoperative hematological indicators for prostate cancer (PCa) patients with laparoscopic radical prostatectomy (LRP) and construct a nomogram prediction model based on hematological indicators and clinicopathological characteristics.

**Method:**

PCa patients who underwent LRP in Beijing Chaoyang Hospital from January 2017 to December 2022 were retrospectively analyzed. Clinicopathological data and blood indicators, including the neutrophil-to-lymphocyte ratio (NLR), platelet-to-lymphocyte ratio (PLR), lymphocyte-to-monocyte ratio (LMR), red blood cell distribution width (RDW), prognosis nutritional index were compared between non-recurrence and recurrence groups. The NPL-IRS score was inflammatory response system score based on the cut-off values NLR, PLR, LMR. Kaplan-Meier analysis was used to calculate the prognostic survival curve. Univariable and multivariable Cox regression risk models were used to identify independent risk factors. A nomogram prediction model was developed, and its accuracy was evaluated and validated through receiver operating characteristic (ROC) curve, C-index, and calibration curve. Internal validation was conducted using Bootstrap method, and the model was also evaluated through external validation.

**Results:**

The number of PCa patients in the training set and external validation set was 210 and 110, respectively. A higher NLR, PLR, RDW, and NPL-IRS score but lower LMR and prognosis nutritional index levels were related to a poor recurrence-free survival (RFS). In training set, the area under the curve (AUC) of the NLR, PLR, LMR, NPL-IRS score, prognosis nutritional index, and RDW were 0.735, 0.710, 0.719, 0.768, 0.728, and 0.599, respectively. Prostate specific antigen density (PSAD), prognosis nutritional index, NPL-IRS score, Gleason score (GS), and positive surgical margin (PSM) were independent risk factors. A new nomogram model was constructed based on these parameters to predict one-year, three-year, and five-year RFS with the AUC of 0.828, 0.867, and 0.892, which could provide an additional clinical net benefit. In external validation set, the AUCs were 0.847, 0.894, and 0.906, respectively.

**Conclusions:**

Higher preoperative NLR, PLR, and RDW or lower LMR and prognosis nutritional index are associated with poorer RFS. The nomogram prediction model based on preoperative PSAD, prognosis nutritional index, NPL-IRS score, GS, and PSM provides important postoperative treatment guidance.

## Introduction

Prostate cancer (PCa) is the world’s second most frequent cancer and the fifth leading cause of cancer death among men in 2022. In China, the incidence of PCa is much lower than in Europe and America, but it has been on the rise year by year in recent years (1, 2). For patients with limited and advanced PCa, robotic-assisted radical prostatectomy (RARP) and laparoscopic radical prostatectomy (LRP) are mature treatments, with the advantages of less trauma and delicate operation, which can prolong life expectancy and improve prognosis. However, due to the complexity of the pelvic structure and the small surgical scope, it is prone to the occurrence of residual tumor tissue or positive margins, which subsequently affects the patient’s prognosis (3). Lymph node metastasis and pathological type are now considered prognostic indicators for PCa, but most can only be evaluated after surgery. It is essential to effectively predict the prognosis of PCa patients and develop individualized treatment plans.

Some common preoperative hematological indices, such as routine blood count and biochemistry results, are easy and quick to obtain and play an important role in suggesting the body’s inflammatory state, immune function, and metabolic level; thus, they have good application value in the prognostic assessment of a variety of solid tumors (4–6).

Alterations in the tumor microenvironment can promote tumorigenesis and tumor development, with systemic inflammation being closely associated with tumors and involved in infiltration and metastasis, as well as treatment prognosis (7). The systematic immune-inflammation index, neutrophil-to-lymphocyte ratio (NLR), platelet-to-lymphocyte ratio (PLR), and lymphocyte-to-monocyte ratio (LMR) are common indicators of systemic inflammation. Studies have shown that a high preoperative NLR is a risk factor for recurrence and poor prognosis in a variety of cancers, including gastric and colorectal cancers (8, 9). H. Lee et al. showed that the cut-off value of the preoperative NLR in PCa was 2.5, and a high NLR was significantly associated with poor pathological outcomes, as well as lower progression-free survival (PFS). Additionally, NLR was an independent predictor of biochemical recurrence (BCR) (10). An elevated PLR also indicates systematic inflammatory and abnormal immune responses and is a risk factor for poor prognosis in malignancies such as breast cancer and non-small cell lung cancer (11, 12). Another study showed that the LMR was negatively correlated with the tumor infiltration depth, stage, grade, size, and Gleason score (13). The red blood cell distribution width (RDW) reflects the heterogeneity of the red blood cell volume. The systemic inflammatory state leads to the release of more immature erythrocytes into the peripheral circulation, increasing peripheral erythrocyte heterogeneity and leading to higher RDW values (14). The prognostic nutritional index, associated with tumor infiltration and metastasis, is a widely used biomarker reflecting pretreatment nutritional status and the systemic immune response (15).

In this study, we developed a prognostic model for PCa patients undergoing LRP by integrating readily available preoperative hematological indices and clinical characteristics. Our goal is to provide clinicians with a practical tool to optimize personalized treatment strategies and improve patient outcomes.

## Materials and methods

### Patients and data collection

This study retrospectively analyzed PCa patients who underwent LRP at Beijing Chaoyang Hospital, Capital Medical University, from January 2017 to December 2022 and used Jiahe electronic medical record system (Beijing Jiahe Meikang Information Technology Co., Ltd., China) to collect demographic data and clinical laboratory indicators to analyze their prognostic predictive value and establish a prediction model. The training set was sourced from the Dongdaqiao Campus of Beijing Chaoyang Hospital, while the external validation dataset was obtained from the Shijingshan Campus during the same period.

The inclusion criteria are as follows: (1) preoperative prostate puncture biopsy and postoperative pathology results indicating PCa; (2) no receipt of radiotherapy and endocrine therapy (such as androgen deprivation therapy) before surgery; (3) no distant metastases, hematological and immune diseases, or bacterial and viral infections; and (4) complete medical records, with routine blood and biochemical test results within 1 week before surgery. At the same time, the regular outpatient follow-up data were expected to be relatively complete. The main exclusion criteria were unresectable tumor, other oncological diseases, and contraindications to surgery.

To assess their general condition, systemic inflammatory status, immuno-nutritional status, and tumor-related characteristics, the following information was collected: general information included age, body mass index (BMI), chronic diseases history. PCa-related information included prostate-specific antigen (PSA), free/total PSA (f/t PSA), prostate volume (PV), prostate-specific antigen density (PSAD), biopsy-positive cores (BPC), Gleason score (GS), perineural invasion, lymphovascular invasion, positive surgical margin (PSM), clinical T stage (cT-stage), and D’Amico risk classification. Perineural invasion was defined as the presence of tumor cells along nerves and/or within the epineurium, perineurium, or endoneurium, where tumor cells encircle at least 33% of the nerve circumference. Lymphovascular invasion was defined as the presence of tumor cells within endothelial-lined luminal spaces of lymphatic vessels. Low-risk PCa was defined as a PSA<10.0 ng/mL, GS <7, and clinical stage T1c to T2a at the initial 10-core biopsy. Intermediate-risk PCa was defined as PSA≥10 ng/mL and <20 ng/mL, GS=7, and clinical stage T2b. High-risk PCa was defined as PSA>20.0 ng/mL, GS >7, and clinical stage≥T2c.

The collected routine blood and biochemical indicators included neutrophil count (×10^9^/L), platelet count (×10^9^/L), lymphocyte count (×10^9^/L), monocyte count (×10^9^/L), RDW (%), and serum albumin concentration (g/L). The NLR, PLR, LMR, and prognosis nutritional index values were obtained according to the following formulae: NLR = neutrophil count/lymphocyte count; PLR = platelet count/lymphocyte count; LMR = lymphocyte count/monocyte count; and prognosis nutritional index = serum albumin concentration + 5 × lymphocyte count. The study was conducted in accordance with the Declaration of Helsinki and was approved by the Ethics Committee of Beijing Chaoyang Hospital (2020-science-299-1).

### Outcome determination and postoperative follow-up

Patients after LRP were divided into non-recurrence and recurrence groups. The recurrence group was defined as postoperative BCR, bone metastasis or distant metastasis, or death. BCR was defined as PSA exceeding 0.2 ng/mL twice consecutively after LRP but without visible recurrent or metastatic lesions on imaging examination. Non-recurrence group was defined as no BCR and radiographic progression by the deadline of follow-up. Recurrence-free survival (RFS) was defined as the time from surgery to BCR or radiographic progression (CT/MRI/bone scan).

All patients were followed up regularly in the outpatient department, and recovery or recurrence was judged by checking the outpatient medical record system and reviewing the results of PSA and other blood tests. Survival and treatment information could also be obtained by telephone from the patients or from a person who knew their current status. The deadline for follow-up was the disease recurrence (BCR/bone metastasis or distant metastasis/death) or December 31, 2023.

### Statistical analysis

Statistical analysis was performed using the SPSS24.0, GraphPad Prism 9.5, and R software. Data conforming to the normal distribution are described in the form of mean ± standard deviation and comparisons between the means of the two groups was performed by the t-test. Numerical data were compared with the Chi-squared test or Fisher’s exact test. GraphPad Prism 9.5 was applied to draw ROC curves, the AUC was calculated to evaluate the predictive efficacy of each index, and the sensitivity, specificity, optimal cutoff value, and Jordon’s index were calculated. The first occurrence of an event with a recurrence was defined as a positive event. The prognostic survival curve was calculated by a Kaplan-Meier analysis, and the significance was compared by the log-rank test. Univariable analysis variables with *P*<0.05 were included in the multivariable Cox regression analysis. The nomogram prediction model was constructed by applying the R language rms package based on the results of multivariable analysis, which was validated using ROC curve, C-index, calibration curves, and decision curves were used to assess its predictive efficacy. Internal validation was conducted using Bootstrap method, and the model was also evaluated through external validation. A two-sided *P*<0.05 was considered a statistically significant difference.

## Results

### Clinicopathologic characteristics and prognosis

**Figure 1:**
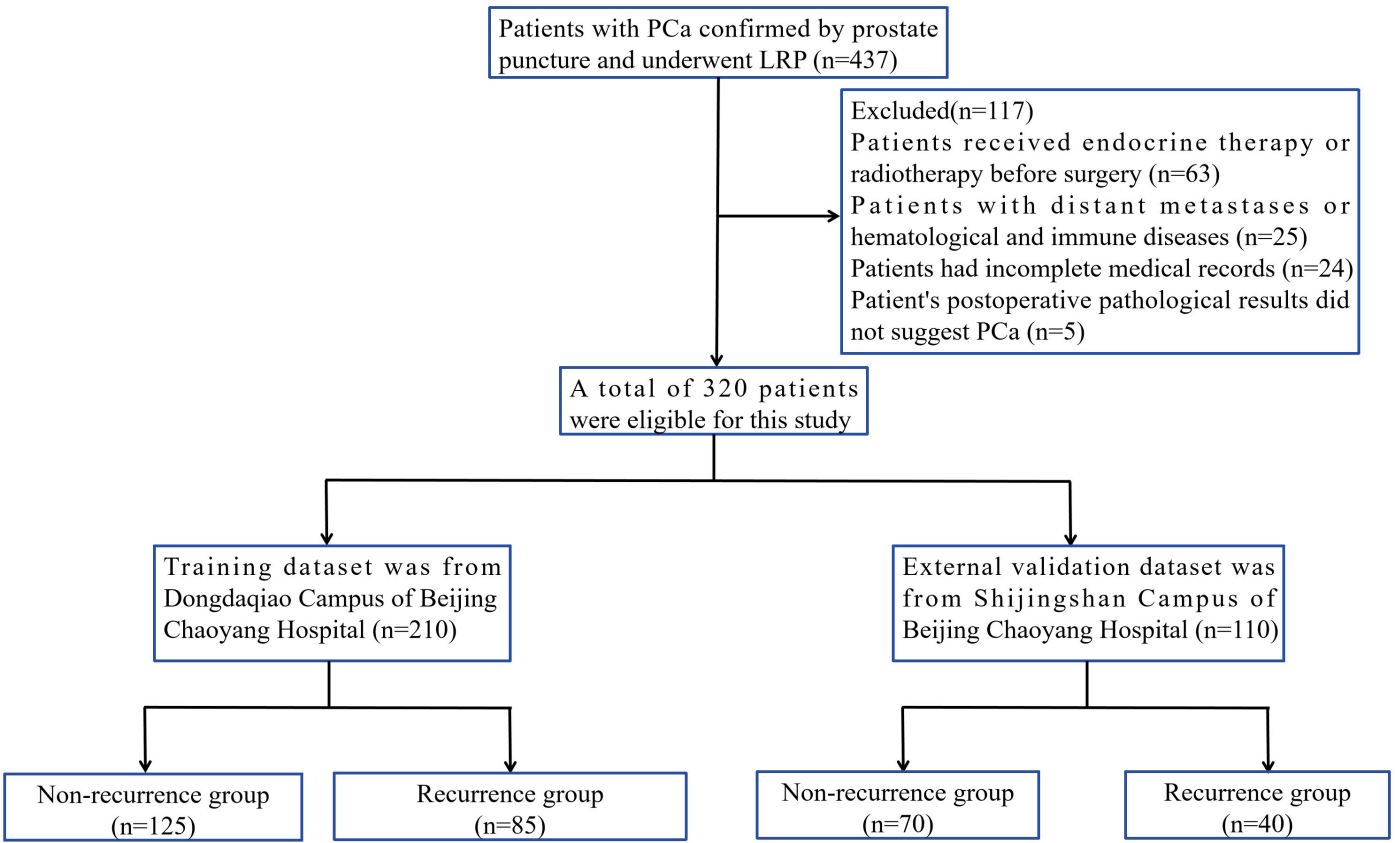
Study flow chart.

A total of 320 PCa patients were assessed, and the number of PCa patients in the training set and external validation set was 210 and 110, respectively (**Figure 1**). In training set, the postoperative follow-up time was 2~83 months, with an mean follow-up of 29.22 months. According to the criteria for determining the prognosis of postoperative patients, the final number of patients in the non-recurrence and recurrence groups were 125 and 85, respectively. In the recurrence group, BCR occurred in 68 cases, and lymphatic metastasis, bone metastasis, and/or distant metastasis or death occurred in 17 cases. Among patients with BCR, patients received postoperative adjuvant endocrine therapy or radiotherapy. In the external validation set, the number of patients with non-recurrence and recurrence group were 70 and 40, with an mean follow-up of 30.56 months. According to **Table 1**, there was no statistically significant difference in clinicopathological data between training and validation sets.

**Table 1:** Comparison of clinicopathological characteristics between training set and external validation set.

Characteristics	Training set (n=210)	External validation set (n=110)	t/χ^2^	*P* value
Age	68.33 ± 6.66	69.21 ± 5.81	1.172	0.242
BMI, kg/m^2^	25.13 ± 2.94	25.66 ± 3.55	1.423	0.156
Preoperative highest PSA, ng/mL	20.20 ± 21.14	19.53 ± 23.77	0.258	0.796
f/t PSA ( $\bar{\text{x}}$ ± SD)	0.13 ± 0.08	0.15 ± 0.06	1.881	0.061
PV, cm^3^	38.11 ± 18.18	40.69 ± 23.28	1.094	0.275
PSAD ( $\bar{\text{x}}$ ± SD), ng/mL^2^	0.60 ± 0.60	0.62 ± 0.76	0.362	0.718
BPC ratio ( $\bar{\text{x}}$ ± SD)	0.47 ± 0.28	0.52 ± 0.29	1.357	0.176
D’Amico risk classification (n, %)			0.265	0.876
Low risk	31 (14.8)	18 (16.4)		
Intermediate risk	70 (33.3)	38 (34.5)		
High risk	109 (51.9)	54 (49.1)		
GS (n, %)			0.071	0.965
≤6	23 (11.0)	11 (10.0)		
7	112 (53.3)	59 (53.6)		
≥8	75 (35.7)	40 (36.4)		
Surgical margin (n, %)			0.828	0.363
Negative	90 (42.9)	53 (48.2)		
Positive	120 (57.1)	57 (51.8)		
Perineural invasion (n, %)			0.000	0.993
Yes	166 (79.0)	87 (79.1)		
No	44 (21.0)	23 (20.9)		
Lymphovascular invasion (n, %)			0.225	0.635
Yes	54 (25.7)	31 (28.2)		
No	156 (74.3)	79 (71.8)		
cT stage (n, %)			3.700	0.054
T2	122 (58.1)	76 (69.1)		
T3~T4	88 (41.9)	34 (30.9)		

The clinicopathological data of the non-recurrence and recurrence groups in training set are shown in **Table 2**. The differences between the two groups in terms of age; BMI; chronic diseases history; f/t PSA; PV; and perineural invasion were not statistically significant (*P*>0.05). However, the highest preoperative PSA (16.51 ± 18.50 ng/mL vs. 25.62 ± 23.60 ng/mL), PSAD (0.46 ± 0.43 ng/mL^2^ vs. 0.79 ± 0.74 ng/mL^2^), BPC ratio (0.43 ± 0.27 vs. 0.54 ± 0.29), D’Amico risk classification (low risk, intermediate risk, high risk, respectively: 18.4%, 40.8%, 40.8% vs. 9.4%, 22.4%, 68.2%), and cT-stage (T2, T3~T4, respectively: 64.0%, 36.0% vs. 49.4%, 50.6%) were significantly different (*P*<0.05). Regarding postoperative pathological data, GS (≤6, 7, ≥8, respectively: 2.4%, 41.2%, 56.4% vs. 16.8%, 60.8%, 22.4%), PSM (80.0% vs. 54.4%), and lymphovascular invasion (35.3% vs. 19.2%) were higher in recurrence group, and the difference was statistically significant (*P*<0.05).

**Table 2:** Comparison of clinicopathological characteristics between non-recurrence and recurrence group in training set.

Characteristics	Total (n=210)	Non-recurrence group (n=125)	Recurrence group (n=85)	t/χ^2^	*P* value
Age	68.33 ± 6.67	69.75 ± 7.03	68.88 ± 6.08	0.993	0.322
BMI, kg/m^2^	25.12 ± 2.94	25.07 ± 2.79	25.19 ± 3.16	0.293	0.770
Smoking history	129 (61.4)	74 (59.2)	55 (64.7)	0.647	0.421
Drinking history	114 (54.3)	69 (55.2)	45 (52.9)	0.104	0.747
Hypertension	82 (39.0)	43 (34.4)	39 (45.9)	2.803	0.094
Diabetes	43 (20.5)	23 (18.4)	20 (23.5)	0.818	0.366
Preoperative highest PSA, ng/mL	20.20 ± 21.14	16.51 ± 18.50	25.62 ± 23.60	3.126	0.002*
f/t PSA ( $\bar{\text{x}}$ ± SD)	0.13 ± 0.08	0.13 ± 0.07	0.13 ± 0.08	0.200	0.841
PV, cm^3^	38.11 ± 18.18	38.24 ± 18.06	37.93 ± 18.45	0.121	0.904
PSAD ( $\bar{\text{x}}$ ± SD), ng/mL^2^	0.59 ± 0.59	0.46 ± 0.43	0.79 ± 0.74	4.039	<0.001*
BPC ratio ( $\bar{\text{x}}$ ± SD)	0.47 ± 0.28	0.43 ± 0.27	0.54 ± 0.29	2.859	0.005*
D’Amico risk classification (n, %)				15.271	<0.001*
Low risk	31 (14.8)	23 (18.4)	8 (9.4)		
Intermediate risk	70 (33.3)	51 (40.8)	19 (22.4)		
High risk	109 (51.9)	51 (40.8)	58 (68.2)		
GS (n, %)				29.556	<0.001*
≤6	23 (11.0)	21 (16.8)	2 (2.4)		
7	111 (52.9)	76 (60.8)	35 (41.2)		
≥8	76 (36.1)	28 (22.4)	48 (56.4)		
Surgical margin (n, %)				14.530	<0.001*
Negative	74 (35.2)	57 (45.6)	17 (20.0)		
Positive	136 (64.8)	68 (54.4)	68 (80.0)		
Perineural invasion (n, %)				1.732	0.188
Yes	166 (79.0)	95 (76.0)	71 (83.5)		
No	44 (21.0)	30 (24.0)	14 (16.5)		
Lymphovascular invasion (n, %)				6.861	0.009*
Yes	54 (25.7)	24 (19.2)	30 (35.3)		
No	156 (74.3)	101 (80.8)	55 (64.7)		
cT stage (n, %)				4.423	0.035*
T2	122 (58.1)	80 (64.0)	42 (49.4)		
T3~T4	88 (41.9)	45 (36.0)	43 (50.6)		

*Means the p-value<0.05 is considered statistically significant.

### Preoperative hematological indices and prognosis

The preoperative hematological indices of the non-recurrence and recurrence groups in training set are shown in **Table 3**. Compared with the non-recurrence group, the NLR (3.18 ± 1.41 vs. 2.23 ± 0.87), PLR (157.54 ± 54.86 vs. 121.68 ± 35.61), and RDW (13.03 ± 0.91% vs. 12.77 ± 0.85%) were significantly higher in the recurrence group (*P*<0.05), while the LMR (3.75 ± 1.60 vs. 4.78 ± 1.53) and prognosis nutritional index (47.02 ± 5.78 vs. 51.55 ± 5.00) were significantly lower (*P*<0.001).

**Table 3:** Comparison of preoperative hematological indices between non-recurrence and recurrence group in training set.

Group	NLR	PLR	LMR	NPL-IRS score	RDW(%)	Prognosis nutritional index
Non-recurrence group (n=125)	2.23 ± 0.87	121.68 ± 35.61	4.78 ± 1.53	1.14 ± 1.07	12.77 ± 0.85	51.55 ± 5.00
Recurrence group (n=85)	3.18 ± 1.41	157.54 ± 54.86	3.75 ± 1.60	2.26 ± 0.98	13.03 ± 0.91	47.02 ± 5.78
t	5.991	5.745	4.692	7.748	2.114	6.047
*P* value	<0.001*	<0.001*	<0.001*	<0.001*	0.036*	<0.001*

*Means the p-value<0.05 is considered statistically significant.

**Figure 2:**
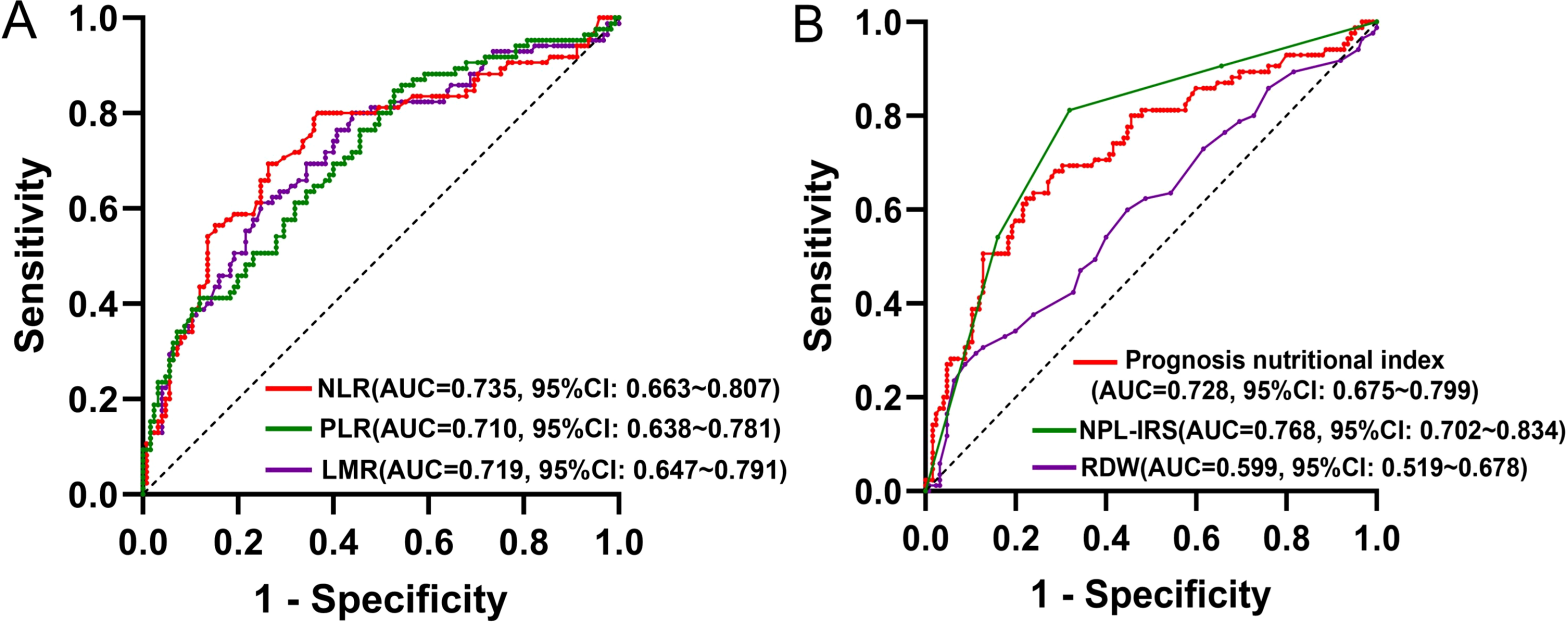
**(A)** ROC curves for NLR, PLR, LMR; **(B)** ROC curves for prognosis nutritional index, NPL-IRS score, RDW.

**Figure 3:**
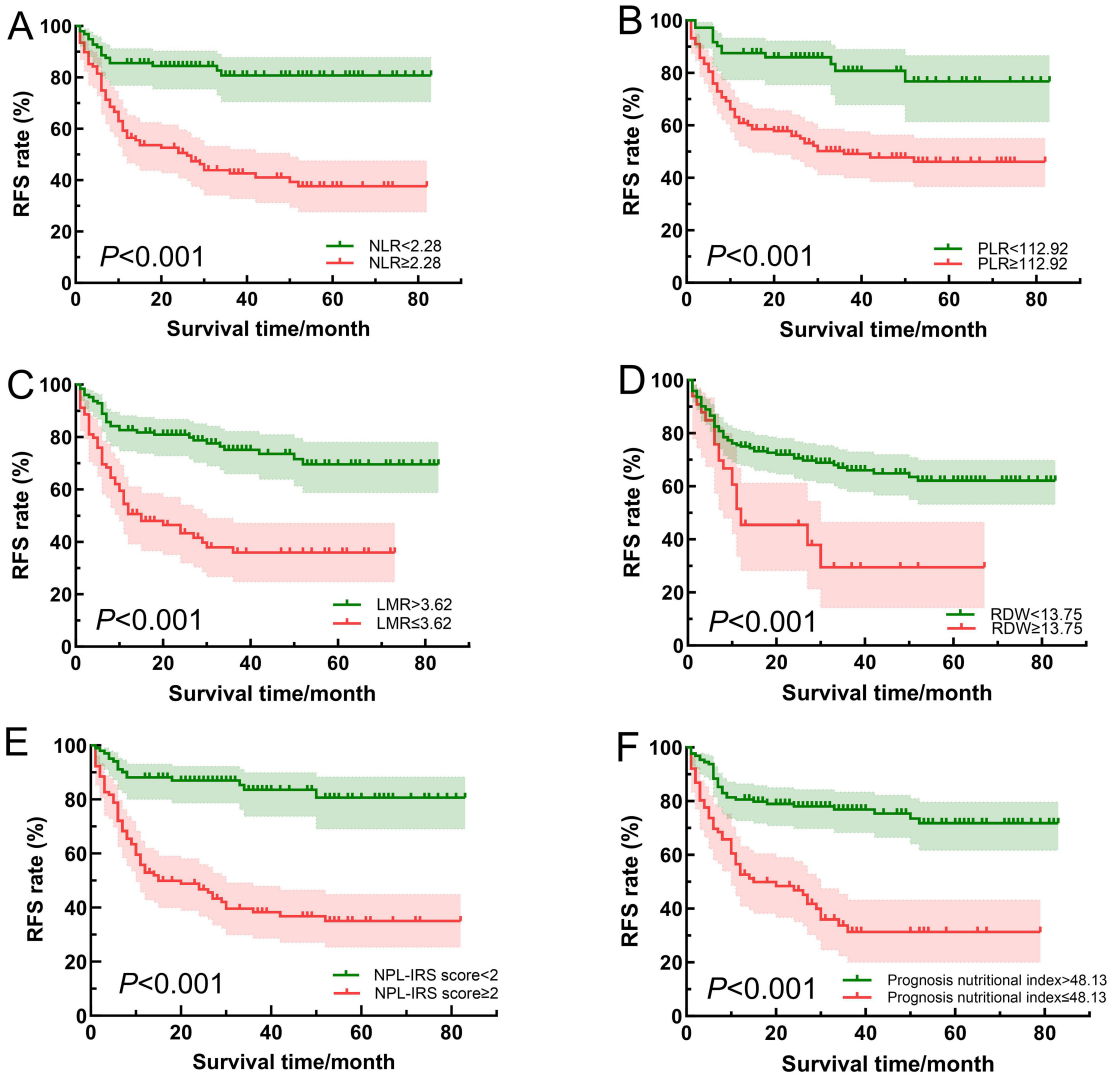
Comparison of survival curves of patients in different NLR **(A)**, PLR **(B)**, LMR **(C)**, RDW **(D)**, NPL-IRS score **(E)** and prognosis nutritional index **(F)** groups.

In training set, ROC curves were analyzed for the NLR, PLR, LMR, prognosis nutritional index, and RDW, and the AUC was calculated to compute the optimal cut-off value based on the Jordon index. The results showed that the AUCs for the NLR, PLR, LMR, prognosis nutritional index, and RDW were 0.735 (95% CI: 0.663~0.807), 0.710 (95% CI: 0.638~0.781), 0.719 (95% CI: 0.647~0.791), 0.728 (95% CI: 0.675~0.799), 0.599 (95% CI: 0.519~0.678), and the cut-off values were 2.28, 112.92, 3.62, 48.13, and 13.75%, respectively, with *P*-values less than 0.05 (**Table 4**, **Figures 2A, B**). The survival analysis results (**Figure 3**) showed that the RFS time of patients with NLR≥2.28, PLR≥112.92, LMR ≤ 3.62, RDW≥13.75% and prognosis nutritional index ≤ 48.13 was significantly lower than that of patients with NLR<2.28, PLR<112.92, LMR>3.62, RDW<13.75% and prognosis nutritional index>48.13 (*P*<0.001).

**Table 4:** Value of preoperative inflammatory indices and prognostic nutritional index in predicting PCa recurrence in training set.

Index	Optimal cut-off value	AUC	AUC95%CI	Sensitivity(%)	Specificity (%)	Youden Index	*P* value
NLR	2.28	0.735	0.663~0.807	80.0	64.0	0.440	<0.001*
PLR	112.92	0.710	0.638~0.781	84.7	47.2	0.319	<0.001*
LMR	3.62	0.719	0.647~0.791	75.2	61.2	0.364	<0.001*
NPL-IRS score	2 point	0.768	0.702~0.834	81.2	68.0	0.492	<0.001*
Prognosis nutritional index	48.13	0.728	0.675~0.799	77.6	62.4	0.400	<0.001*
RDW	13.75%	0.599	0.519~0.678	27.1	91.2	0.183	0.015*

*Means the P-value<0.05 is considered statistically significant.

### High NPL-IRS score was associated with poor RFS

To comprehensively assess the systemic inflammatory status of the PCa patients, the NPL-IRS score was constructed based on the optimal cut-off values of the NLR, PLR, and LMR (**Table 5**). As shown in **Table 3**, the NPL-IRS score in the recurrence group was significantly higher than that in the non-recurrence group (2.26 ± 0.98 vs. 1.14 ± 1.07). According to **Table 4**; **Figure 2B**, ROC curve analysis showed that the AUC of the NPL-IRS score in predicting patients’ poor RFS was 0.768 (95% CI: 0.702~0.834), with an optimal cut-off value of 2 points (sensitivity 81.2%, specificity 68.0%). As shown in **Figure 3**, NPL-IRS score ≥2 points was associated with significantly shorter RFS (P<0.001).

**Table 5:** Criteria for the construction of the NPL-IRS score system.

Criteria	NPL-IRS score
Satisfy all conditions of NLR ≤ 2.28, PLR ≤ 112.92, LMR>3.62	0
Satisfy 1 of NLR > 2.28, PLR > 112.92, LMR ≤ 3.62	1
Satisfy 2 of NLR > 2.28, PLR > 112.92, LMR ≤ 3.62	2
Satisfy all conditions of NLR>2.28, PLR>112.92, LMR ≤ 3.62	3

### High NPL-IRS scores and low prognosis nutritional index were correlated with adverse pathologic characteristics

In the training set, patients were stratified by NPL-IRS scores (<2 points and ≥2 points). As shown in **Supplementary Table S1**, no significant differences in BMI, preoperative maximum PSA, f/t PSA, PV, PSAD, BPC ratio, perineural invasion, lymphovascular invasion and cT-stage were observed between groups (P>0.05). However, the NPL-IRS≥2 group had significantly older age, higher D’Amico risk classification and GS, as well as increased PSM rates (P<0.05). Patients with prognosis nutritional index <48.13 and ≥48.13 were analyzed with the same method, and the results are shown in **Supplementary Table S2**. The results revealed significant differences in age, BPC ratio, D’Amico risk classification, GS, and PSM (*P*<0.05).

### Preoperative PSAD, prognosis nutritional index, NPL-IRS score, GS and PSM were independent predictors of prognosis

In the training set, taking the prognosis of patients as the dependent variable (0=non-recurrence, 1=recurrence, and t=time from operation to recurrence), age, BMI, highest preoperative PSA, f/t PSA, PV, PSAD, BPC ratio, risk classification (0=low risk, 1=intermediate risk, and 2=high risk), NLR, PLR, LMR, RDW, NPL-IRS score, prognosis nutritional index, GS (0=GS ≤ 6, 1=GS=7, and 2=GS≥8), cT-stage (0=T2 and 1=T3~T4), perineural invasion (0=none and 1=yes), lymphovascular invasion (0=none and 1=yes), and surgical margin (0=negative and 1=positive) were subjected to univariable Cox regression analysis (**Table 6**). Variables from the univariable analyses with *P*<0.05 were included in the multivariable Cox regression analysis, which showed that preoperative PSAD, prognosis nutritional index, NPL-IRS score, GS, and PSM were significantly associated with the postoperative recurrence of PCa patients (*P*<0.05).

**Table 6:** Univariable and multivariable Cox regression analysis of prognosis in PCa patients in training set.

Characteristics	Univariable analysis			Multivariable analysis		
	Hazard ratio	95%CI	*P* value	Hazard ratio	95%CI	*P* value
Age	1.017	0.985~1.049	0.310			
BMI	1.003	0.930~1.083	0.933			
Preoperative highest PSA	1.010	1.003~1.017	0.007*	0.991	0.977~1.005	0.200
f/t PSA	0.999	0.973~1.025	0.914			
PV	0.999	0.988~1.011	0.916			
PSAD	1.542	1.209~1.967	<0.001*	1.776	1.038~3.005	0.036*
BPC ratio	1.011	1.004~1.019	0.003*	0.995	0.986~1.005	0.339
D’Amico risk classification						
Low risk (Reference)	1.000			1.000		
Intermediate risk	1.073	0.469~2.451	0.868	0.584	0.240~1.419	0.235
High risk	2.629	1.254~5.513	0.010*	0.615	0.249~1.520	0.293
NLR	1.404	1.252~1.575	<0.001*	1.024	0.812~1.292	0.840
PLR	1.010	1.007~1.014	<0.001*	0.998	0.992~1.004	0.512
LMR	0.663	0.557~0.789	<0.001*	1.117	0.897~1.392	0.322
RDW	1.267	1.013~1.585	0.038*	0.759	0.574~1.004	0.053
NPL-IRS score	2.018	1.621~2.513	<0.001*	1.834	1.247~2.669	0.002*
Prognosis nutritional index	0.887	0.854~0.922	<0.001*	0.929	0.884~0.976	0.004*
GS						
≤6 (Reference)	1.000			1.000		
7	4.422	1.064~18.373	0.041*	2.886	0.654~12.902	0.165
≥8	11.204	2.716~46.228	0.001*	6.567	1.338~32.238	0.020*
Perineural invasion	1.458	0.822~2.586	0.198			
Lymphovascular invasion	1.947	1.246~3.043	0.003*	1.136	0.667~1.933	0.639
PSM	4.135	2.426~7.047	<0.001*	2.701	1.500~4.864	0.001*
cT stage	1.766	1.151~2.707	0.009*	1.278	0.731~2.234	0.390

*Means the p-value<0.05 is considered statistically significant.

### Development and validation of a novel nomogram to predict postoperative prognosis of PCa patients

**Figure 4:**
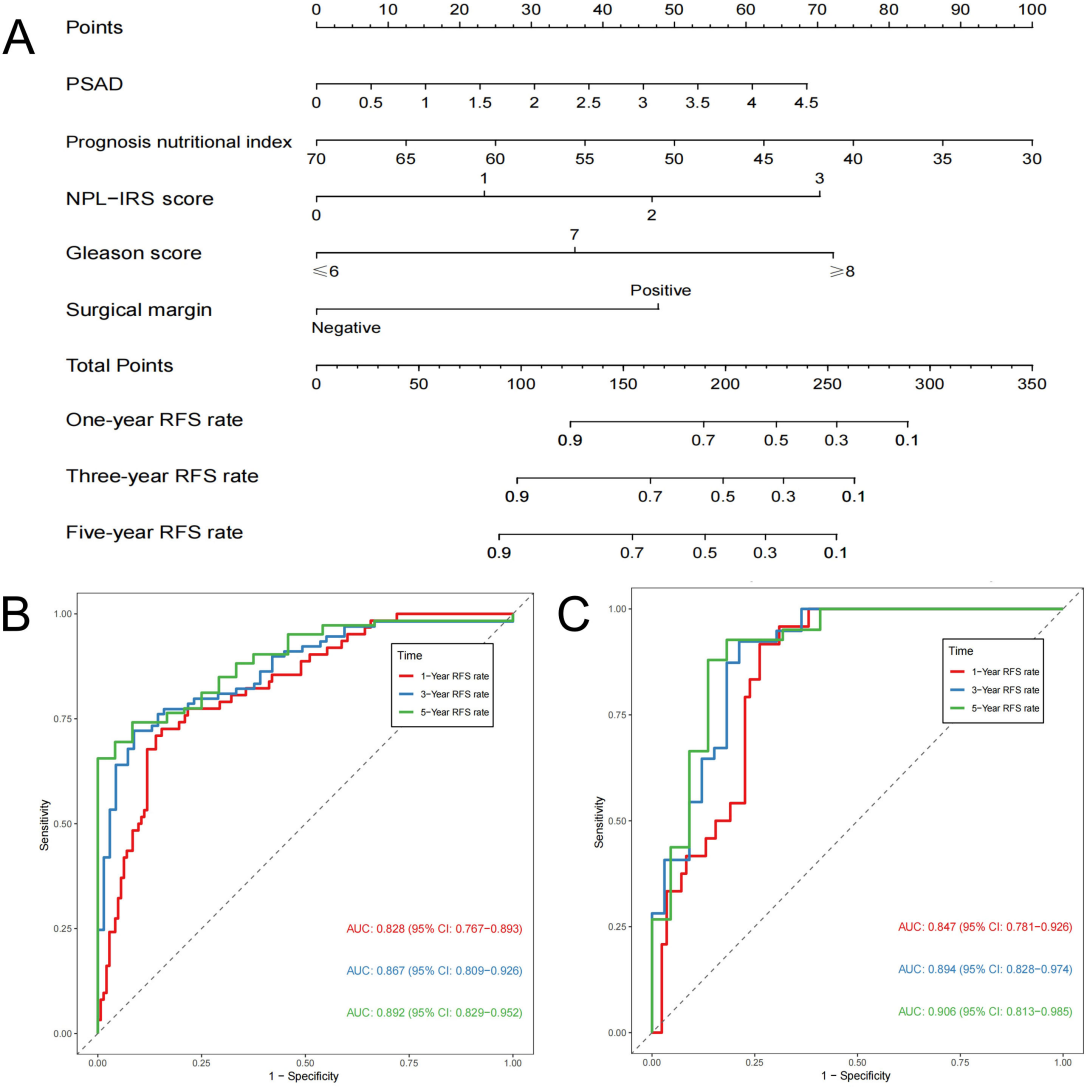
Nomogram prediction model for postoperative prognosis of patients with prostate cancer **(A)**; ROC curves of training set **(B)**; ROC curves of external verification data set **(C)**.

**Figure 5:**
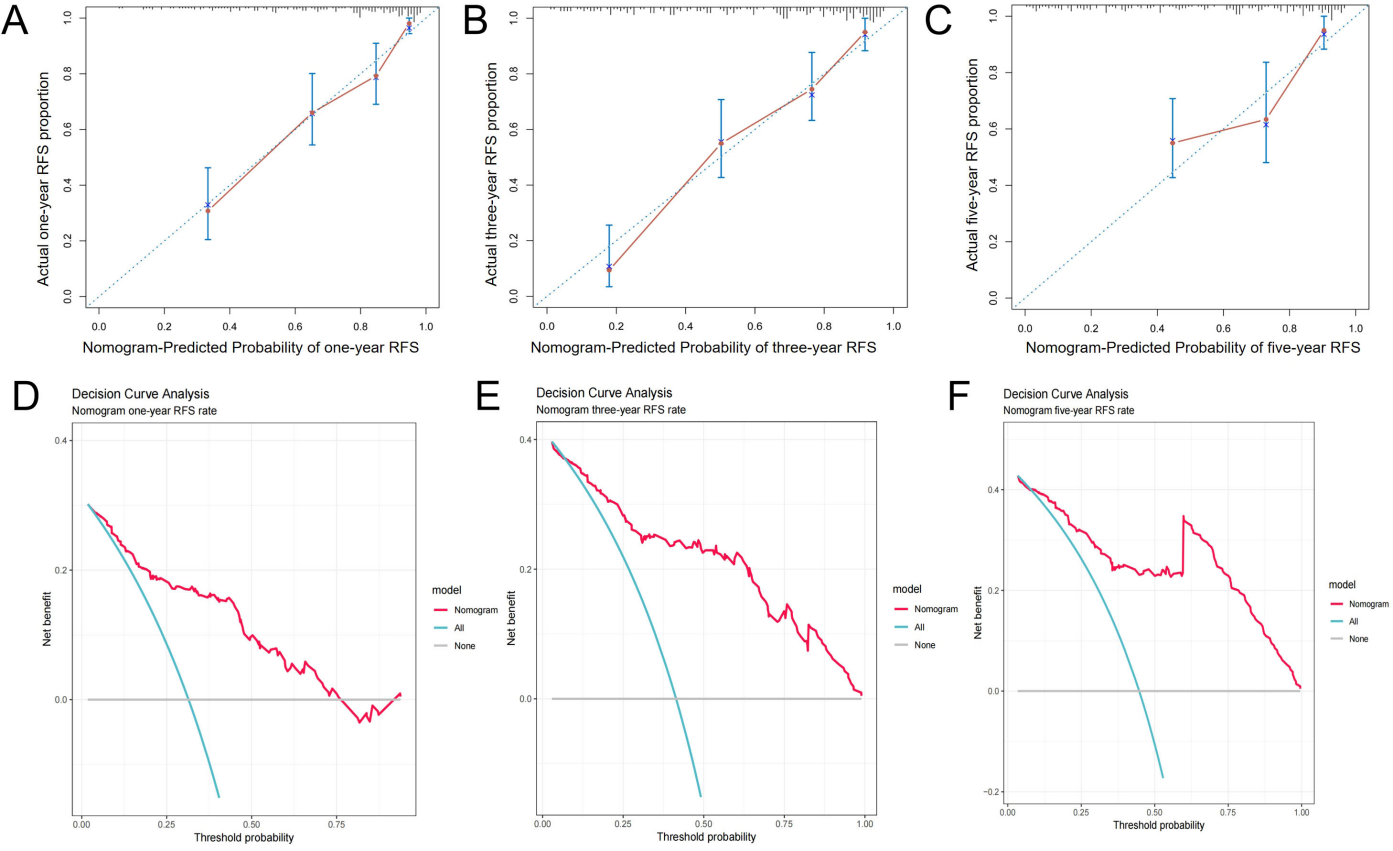
Calibration curves **(A-C)**, and decision curves **(D-F)** of the prognostic prediction model of training set.

A nomogram prediction model for predicting the postoperative prognosis of PCa patients was constructed (**Figure 4A**). Bootstrap method was used to conduct internal validation, the AUCs of the nomogram model for predicting one-year, three-year, and five-year RFS rate were verified to be 0.828 (95% CI: 0.767~0.893), 0.867 (95% CI: 0.809~0.926), and 0.892 (95% CI: 0.829~0.952) (**Figure 4B**), suggesting that this model has good prediction accuracy. As shown in **Figures 5A–F**, the calibration curves are close to the ideal curves and decision curves analysis showed that the predictive model had good clinical applicability. The external verification results showed that the C-index of the prediction model was 0.821 (95% CI: 0.765~0.877). The AUCs for predicting one-year, three-year, and five-year RFS rate were 0.847 (95% CI: 0.781~0.926), 0.894 (95% CI: 0.828~0.974), and 0.906 (95% CI: 0.813~0.985), respectively (**Figure 4C**). After internal and external validation, the prognostic nomogram for PCa patients developed in this study had demonstrated high predictive accuracy. The abbreviations in this article can be found in **Table 7**.

**Table 7:** Full text abbreviation vocabulary list.

Abbreviation	Full name
AUC	area under the curve
BCR	biochemical recurrence
BMI	body mass index
BPC	biopsy positive cores
CI	confidence interval
CPRC	castration-resistant prostate cancer
cT stage	clinical T stage
GS	Gleason score
HR	hazard ratio
LMR	lymphocyte-to-monocyte ratio
LRP	laparoscopic radical prostatectomy
NLR	neutrophil-to-lymphocyte ratio
NPL-IRS score	inflammatory response system score based on NLR, PLR, LMR
OS	overall survival
PCa	prostate cancer
PFS	progression-free survival
PLR	platelet-to-lymphocyte ratio
PSA	prostate specific antigen
PSAD	prostate specific antigen density
PSM	positive surgical margin
PV	prostate volume
RFS	recurrence-free survival
RDW	red blood cell distribution width
ROC	receiver operating characteristic

## Discussion

### Hematological markers

It was reported that many factors affect the prognosis of PCa after LRP, and several studies have shown that a high preoperative PSA level, a high postoperative pathological GS and tumor stage, PSM, lymph node invasion, and perineural invasion are independent risk factors for BCR (16–20). The above findings are similar to the results of our study, in which the recurrence group had higher PSA, PSAD, GS, BPC, and cT-stage, with the highest preoperative PSA, PSAD, and GS being independent risk factors for poor RFS.

In PCa, elevated NLR and PLR, along with reduced LMR, have been shown to correlate strongly with a higher T-stage, GS, and distant metastases (13, 21, 22). Our findings were similar to those described above, demonstrating that higher tumor stage and GS correlate with increased NLR, PLR or decreased LMR. Moreover, NLR, PLR, and LMR exhibited comparable efficacy in predicting reduced RFS, with AUCs of 0.735, 0.710, and 0.719, respectively. Consistently, survival analysis confirmed significantly worse RFS in PCa patients with elevated NLR, PLR or diminished LMR. Joanna Huszno et al. analyzed the correlation between the hematological parameters of 152 PCa patients and their prognosis, which showed that elevated NLR, platelets, neutrophils, and leukocytes were significantly correlated with reduced overall survival (OS) (23). Li F et al. revealed PLR is significantly increased in PCa patients, and it is an independent predictor of 3-year mortality (22). To reflect the state of the tumor’s immune environment and the level of body inflammation, we established the NPL-IRS score system using optimal NLR, PLR, and LMR cutoff values. Our analysis showed that the NPL-IRS scores of the recurrence group were significantly higher, with an AUC of 0.768 and an optimal cut-off value of 2. Patients with NPL-IRS scores ≥2 exhibited advanced age, higher tumor stage, elevated GS, and increased PSM rates. This may be attributed to the strong activation of the inflammatory response and high degree of tumor malignancy in advanced PCa cases, whereas a high T-stage and GS are risk factors for postoperative PSM (24).

The RDW is a parameter reflecting the heterogeneity of red blood cell size, which is mainly used clinically to diagnose anemia. Elevated RDW levels are associated with systemic inflammation, poor nutritional status, and physiological stress (14). Mechanistically, inflammation suppresses erythropoietin-mediated bone marrow stimulation, impairs erythrocyte maturation, and releases immature erythrocytes into peripheral circulation, thereby increasing erythrocyte heterogeneity and elevating RDW (25). Wataru Fukuokaya et al. demonstrated that in PCa patients receiving endocrine therapy, high RDW correlated significantly with lymph node metastasis, visceral metastasis, anemia, hypoalbuminemia, and elevated C-reactive protein. Furthermore, multivariable analysis identified high RDW as an independent predictor of reduced PFS and overall survival (OS) (26). Our study corroborates these findings, revealing significantly higher recurrence and metastasis risks in patients with elevated RDW. However, the predictive efficacy of RDW was poor with an AUC of only 0.599 and a cut-off value of 13.75% in this study.

Prognostic nutritional index, calculated from peripheral blood lymphocyte counts and serum albumin levels, is a validated measure of systemic immunonutritional status. Initially developed for gastrointestinal cancer assessment (27), prognosis nutritional index reflects malnutrition-induced immune compromise that promotes tumor progression. A PCa-focused meta-analysis confirmed that pretreatment prognosis nutritional index significantly predicts survival, with lower baseline values correlating with reduced OS and PFS (15). In hormone-sensitive PCa patients, the prognosis nutritional index also showed good efficacy in predicting prognostic value after endocrine therapy. A study showed that the prognosis nutritional index was an independent prognostic indicator for PFS and OS and that adding the prognosis nutritional index to the prediction model would improve the predictive accuracy. A high pretreatment prognosis nutritional index is a favorable prognostic indicator for PCa patients treated with endocrine therapy (28). The present study reached a similar conclusion that patients with lower prognosis nutritional index had worse RFS, and it was significantly associated with higher BPC ratio, D’Amico risk classification, GS, and PSM.

### Model efficacy

Current prognostic models for PCa patients predominantly rely on clinicopathological features, with limited integration of preoperative hematological markers (29–31). Beyond validating established clinicopathological associations, this study comprehensively evaluated the prognostic impact of systemic inflammatory indices (NLR, PLR, LMR, and RDW), immunonutritional markers (prognostic nutritional index), and the novel NPL-IRS score. Multivariable Cox regression identified preoperative PSAD, prognostic nutritional index, NPL-IRS score, GS, and PSM as independent predictors. The AUCs of the prediction model predicting one-year, three-year, and five-year RFS were 0.828, 0.867, and 0.892, which could provide an additional clinical net benefit. In external validation set, the AUCs were 0.847, 0.894, and 0.906, respectively, suggesting the model has strong predictive efficacy. Therefore, the prediction model constructed in this study is reliable and can quantify the risk of poor postoperative prognosis in PCa patients.

### Limitations

This study also has some limitations. First, it was a single-center, retrospective study with selection bias. Moreover, recall bias due to distorted or incomplete memories during the follow-up process was difficult to avoid. We minimized bias as much as possible by collecting objective data, conducting structured follow-up, and ensuring data quality control. Secondly, while RARP has become the predominant minimally invasive technique, our study utilized LRP due to its widespread availability in participating centers. As a result, the lack of RARP data may limit the generalizability of this model. Third, the study’s sample size was small, although the predictive model showed good predictive efficacy. Therefore, further large-scale prospective studies are needed to validate the model to enhance its generalizability.

## Conclusion

Patients with a poor RFS after LRP had higher preoperative PSA, PSAD, BPC, NLR, PLR, and RDW values and lower LMR and prognosis nutritional index values. Regarding pathological features, those with a high GS, D’Amico risk classification, cT-stage, PSM, and vascular invasion had a poor prognosis. The NLR, PLR, LMR, NPL-IRS score, prognosis nutritional index, and RDW all showed predictive efficacy in PCa patients postoperatively, with the NPL-IRS score having the best predictive value, followed by the prognosis nutritional index. Constructing a predictive model for postoperative prognosis based on the preoperative PSAD, prognosis nutritional index, NPL-IRS score, GS, and PSM has good clinical application value and can provide important guidance for urologists in assessing prognosis and individualized postoperative management.

## Data Availability

The raw data supporting the conclusions of this article will be made available by the authors, without undue reservation.

## References

[1] Bill-Axelson A Holmberg L Garmo H Taari K Busch C Nordling S. Radical prostatectomy or watchful waiting in prostate cancer - 29-year follow-up. N Engl J Med. (2018) 379:2319–29. doi: 10.1056/NEJMoa1807801, PMID: 30575473

[2] Chen J He L Ni Y Yu F Zhang A Wang X. Prevalence and associated risk factors of prostate cancer among a large Chinese population. Sci Rep. (2024) 14:26338. doi: 10.1038/s41598-024-77863-z, PMID: 39487298 PMC11530631

[3] Zhang L Wu B Zha Z Zhao H Jiang Y Yuan J. Positive surgical margin is associated with biochemical recurrence risk following radical prostatectomy: a meta-analysis from high-quality retrospective cohort studies. World J Surg Oncol. (2018) 16:124. doi: 10.1186/s12957-018-1433-3, PMID: 29970100 PMC6029044

[4] Li J Zhu S Tong J Hao H Yang J Liu Z. Suppression of lactate dehydrogenase A compromises tumor progression by downregulation of the Warburg effect in glioblastoma. Neuroreport. (2016) 27:110–5. doi: 10.1097/WNR.0000000000000506, PMID: 26694942 PMC4712768

[5] Zapala P Garbas K Lewandowski Z Slusarczyk A Slusarczyk C Mielczarek L. Neutrophil-to-lymphocyte ratio predicts nodal involvement in unfavourable, clinically nonmetastatic prostate cancer patients and overall survival in pN1 patients. Sci Rep. (2023) 13:392. doi: 10.1038/s41598-023-27542-2, PMID: 36624246 PMC9829873

[6] Liao DW Hu X Wang Y Yang ZQ Li X. C-reactive protein is a predictor of prognosis of prostate cancer: A systematic review and meta-analysis. Ann Clin Lab Sci. (2020) 50:161–71., PMID: 32366552

[7] Mantovani A Allavena P Sica A Balkwill F. Cancer-related inflammation. Nature. (2008) 454:436–44. doi: 10.1038/nature07205, PMID: 18650914

[8] Yamanaka T Matsumoto S Teramukai S Ishiwata R Nagai Y Fukushima M. The baseline ratio of neutrophils to lymphocytes is associated with patient prognosis in advanced gastric cancer. Oncology. (2007) 73:215–20. doi: 10.1159/000127412, PMID: 18424885

[9] Walsh SR Cook EJ Goulder F Justin TA Keeling NJ. Neutrophil-lymphocyte ratio as a prognostic factor in colorectal cancer. J Surg Oncol. (2005) 91:181–4. doi: 10.1002/jso.20329, PMID: 16118772

[10] Lee H Jeong SJ Hong SK Byun SS Lee SE Oh JJ. High preoperative neutrophil-lymphocyte ratio predicts biochemical recurrence in patients with localized prostate cancer after radical prostatectomy. World J Urol. (2016) 34:821–7. doi: 10.1007/s00345-015-1701-6, PMID: 26449784

[11] Koh CH Bhoo-Pathy N Ng KL Jabir RS Tan GH See MH. Utility of pre-treatment neutrophil-lymphocyte ratio and platelet-lymphocyte ratio as prognostic factors in breast cancer. Br J Cancer. (2015) 113:150–8. doi: 10.1038/bjc.2015.183, PMID: 26022929 PMC4647546

[12] Kang KH Efird JT Sharma N Yang M Dowlati A Linden P. Prognostic potential of neutrophil-to-lymphocyte ratio and lymphocyte nadir in stage III non-small-cell lung cancer. Future Oncol. (2017) 13:1405–14. doi: 10.2217/fon-2017-0045, PMID: 28685599

[13] Wang Q Ou T Li J Cui X Liang J. Research on the association of plasma TGF-beta1 level and blood lymphocyte/monocyte ratio with pathological grade, clinical stage and prognosis of prostate cancer. J Buon. (2020) 25:2418–23., PMID: 33277864

[14] Salvagno GL Sanchis-Gomar F Picanza A Lippi G. Red blood cell distribution width: A simple parameter with multiple clinical applications. Crit Rev Clin Lab Sci. (2015) 52:86–105. doi: 10.3109/10408363.2014.992064, PMID: 25535770

[15] Zheng Y Wang K Ou Y Hu X Wang Z Wang D. Prognostic value of a baseline prognostic nutritional index for patients with prostate cancer: a systematic review and meta-analysis. Prostate Cancer Prostatic Dis. (2024) 27:604–13. doi: 10.1038/s41391-023-00689-9, PMID: 37391595

[16] Song W Lee DH Jeon HG Jeong BC Seo SI Lee HM. Impact of Gleason score on biochemical recurrence in patients with pT3aN0/Nx prostate cancer with positive surgical margins: a multicenter study from the Prostate Cancer Research Committee. J Cancer Res Clin Oncol. (2017) 143:2393–400. doi: 10.1007/s00432-017-2502-7, PMID: 28823006 PMC11819247

[17] Panunzio A Sorce G Hoeh B Hohenhorst L Tappero S Nimer N. Effect of positive surgical margins at radical prostatectomy on cancer-specific mortality in high/very high-risk prostate cancer patients with Gleason Grade Group 4-5. Prostate. (2023) 83:268–76. doi: 10.1002/pros.24458, PMID: 36336728

[18] Preisser F Heinze ASAR Budaus L Chun FK Graefen M. Impact of positive surgical margin length and Gleason grade at the margin on oncologic outcomes in patients with nonorgan-confined prostate cancer. Prostate. (2022) 82:949–56. doi: 10.1002/pros.24341, PMID: 35344221

[19] Reeves FA Battye S Roth H Peters JS Hovens C Costello AJ. Prostatic nerve subtypes independently predict biochemical recurrence in prostate cancer. J Clin Neurosci. (2019) 63:213–9. doi: 10.1016/j.jocn.2019.01.052, PMID: 30772200

[20] Tilki D Mandel P Karakiewicz PI Heinze A Huland H Graefen M. The impact of very high initial PSA on oncological outcomes after radical prostatectomy for clinically localized prostate cancer. Urol Oncol. (2020) 38:379–85. doi: 10.1016/j.urolonc.2019.12.027, PMID: 32001198

[21] Gokce MI Tangal S Hamidi N Suer E Ibis MA Beduk Y. Role of neutrophil-to-lymphocyte ratio in prediction of Gleason score upgrading and disease upstaging in low-risk prostate cancer patients eligible for active surveillance. Can Urol Assoc J. (2016) 10:E383–7. doi: 10.5489/cuaj.3550, PMID: 28096923 PMC5234405

[22] Li F Hu H Gu S Chen X Sun Q. Platelet to lymphocyte ratio plays an important role in prostate cancer’s diagnosis and prognosis. Int J Clin Exp Med. (2015) 8:11746–51., PMID: 26380014 PMC4565397

[23] Huszno J Kolosza Z Mrochem-Kwarciak J Telka E Jochymek B Miszczyk L. Role of neutrophil-lymphocyte ratio, platelet-lymphocyte ratio, lymphocyte-monocyte ratio and platelets in prognosis of patients with prostate cancer. Oncol Lett. (2022) 24:305. doi: 10.3892/ol.2022.13425, PMID: 35949621 PMC9353225

[24] Pooli A Salmasi A Johnson DC Lenis AT Faiena I Lebacle C. Positive surgical margins at radical prostatectomy in the United States: Institutional variations and predictive factors. Urol Oncol. (2020) 38:1–17. doi: 10.1016/j.urolonc.2019.08.016, PMID: 31537483

[25] Antic J Jokic R Bukarica S Lukic I Dobrijevic D Rakic G. Predictive value of red blood cell distribution width, mean platelet volume and platelet distribution width in children with acute appendicitis. Children (Basel). (2021) 8:1041. doi: 10.3390/children8111041, PMID: 34828754 PMC8619955

[26] Fukuokaya W Kimura T Onuma H Mori K Honda M Inaba H. Red cell distribution width predicts prostate-specific antigen response and survival of patients with castration-resistant prostate cancer treated with androgen receptor axis-targeted agents. Clin Genitourin Cancer. (2019) 17:223–30. doi: 10.1016/j.clgc.2019.04.010, PMID: 31080022

[27] Buzby GP Mullen JL Matthews DC Hobbs CL Rosato EF. Prognostic nutritional index in gastrointestinal surgery. Am J Surg. (1980) 139:160–7. doi: 10.1016/0002-9610(80)90246-9 7350839

[28] Li B Lu Z Wang S Hou J Xia G Li H. Pretreatment elevated prognostic nutritional index predicts a favorable prognosis in patients with prostate cancer. BMC Cancer. (2020) 20:361. doi: 10.1186/s12885-020-06879-1, PMID: 32349713 PMC7191702

[29] Li S Cai S Huang J Li Z Shi Z Zhang K. Develop prediction model to help forecast advanced prostate cancer patients’ prognosis after surgery using neural network. Front Endocrinol (Lausanne). (2024) 15:1293953. doi: 10.3389/fendo.2024.1293953, PMID: 38577575 PMC10991752

[30] Parr H Porta N Tree AC Dearnaley D Hall E. A personalized clinical dynamic prediction model to characterize prognosis for patients with localized prostate cancer: analysis of the CHHiP phase 3 trial. Int J Radiat Oncol Biol Phys. (2023) 116:1055–68. doi: 10.1016/j.ijrobp.2023.02.022, PMID: 36822374

[31] Wang Z Dai Z Gao Y Zhao Z Li Z Wang L. Development of a machine learning-based predictive risk model combining fatty acid metabolism and ferroptosis for immunotherapy response and prognosis in prostate cancer. Discov Oncol. (2025) 16:744. doi: 10.1007/s12672-025-02484-5, PMID: 40355680 PMC12069205

