# Supplementary material for: Prognostic value and predictive model construction for patients undergoing laparoscopic radical prostatectomy based on the preoperative NPL-IRS score and prognostic nutritional index
Source: Front Oncol. 2025 Aug 20;15:1603993. doi: 10.3389/fonc.2025.1603993 (PMC12404917; doi:10.3389/fonc.2025.1603993)
Supplement: Supplementary file 1 [file Table1.docx]

**Supplementary Table 1** Correlation analysis of patients with NPL-IRS <2 points and NPL-IRS ≥2 points with clinicopathological features

| Characteristics | NPL-IRS＜2 (n=101) | NPL-IRS**≥**2 (n=109) | t/χ^2^ | *P* value |
| --- | --- | --- | --- | --- |
| Age | 67.10±6.81 | 69.47±6.34 | 2.610 | 0.010* |
| BMI, kg/m^2^ | 24.98±2.97 | 25.25±2.91 | 0.643 | 0.521 |
| Preoperative highest PSA, ng/mL | 18.07±17.62 | 22.17±23.86 | 1.408 | 0.160 |
| f/t PSA(‾x±SD) | 0.12±0.09 | 0.14±0.08 | 1.005 | 0.316 |
| PV, cm^3^ | 38.20±17.37 | 38.03±18.97 | 0.069 | 0.945 |
| PSAD (‾x±SD), ng/mL^2^ | 0.56±0.64 | 0.63±0.55 | 0.937 | 0.350 |
| BPC ratio (‾x±SD) | 0.44±0.26 | 0.50±0.30 | 1.661 | 0.098 |
| D'Amico risk classification (n, %) |  |  | 8.451 | 0.015* |
| Low risk | 19(18.8) | 12(11.0) |  |  |
| Intermediate risk | 40(39.6) | 30(27.5) |  |  |
| High risk | 42(41.6) | 67(61.5) |  |  |
| GS (n, %) |  |  | 14.531 | 0.001* |
| ≤6 | 18(17.8) | 5(4.6) |  |  |
| 7 | 57(56.4) | 55(50.5) |  |  |
| ≥8 | 26(25.8) | 49(44.9) |  |  |
| Surgical margin (n, %) |  |  | 4.078 | 0.043* |
| Negative | 64(63.4) | 83(76.1) |  |  |
| Positive | 37(36.6) | 26(23.9) |  |  |
| Perineural invasion (n, %) |  |  | 0.928 | 0.335 |
| Yes | 77(76.2) | 89(81.7) |  |  |
| No | 24(23.8) | 20(18.3) |  |  |
| Lymphovascular invasion (n, %) |  |  | 3.561 | 0.059 |
| Yes | 20(19.8) | 34(31.2) |  |  |
| No | 81(80.2) | 75(68.8) |  |  |
| cT stage (n, %) |  |  | 0.008 | 0.928 |
| T2 | 59(58.4) | 63(57.8) |  |  |
| T3~T4 | 42(41.6) | 46(42.2) |  |  |

*means the p-value<0.05 is considered statistically significant; BMI, body mass index；PSA, prostate specific antigen; PV, prostate volume; PSAD, prostate specific antigen density；BPC, biopsy positive cores; GS, Gleason score, cT stage, clinical T stage.

**Supplementary Table 2** Correlation analysis of patients with prognosis nutritional index＜48.13 and ≥48.13 with clinicopathological features

| Characteristics | Prognosis nutritional index＜48.13  (n=81) | Prognosis nutritional index≥48.13  (n=129) | t/χ^2^ | *P* value |
| --- | --- | --- | --- | --- |
| Age | 70.01±6.26 | 67.27±6.71 | 2.956 | 0.003* |
| BMI, kg/m^2^ | 25.06±3.01 | 25.16±2.90 | 0.247 | 0.805 |
| Preoperative highest PSA, ng/mL | 22.88±24.46 | 18.51±18.67 | 1.459 | 0.146 |
| f/t PSA(‾x±SD) | 0.13±0.08 | 0.13±0.09 | 1.101 | 0.272 |
| PV, cm^3^ | 38.64±18.94 | 37.78±17.74 | 0.335 | 0.738 |
| PSAD (‾x±SD), ng/mL^2^ | 0.67±0.60 | 0.55±0.59 | 1.361 | 0.175 |
| BPC ratio (‾x±SD) | 0.53±0.30 | 0.44±0.26 | 2.303 | 0.022* |
| D'Amico risk classification (n, %) |  |  | 7.984 | 0.018* |
| Low risk | 9(11.1) | 22(17.1) |  |  |
| Intermediate risk | 20(24.7) | 50(38.8) |  |  |
| High risk | 52(64.2) | 57(44.1) |  |  |
| GS (n, %) |  |  | 9.907 | 0.007* |
| ≤6 | 5(6.2) | 18(14.0) |  |  |
| 7 | 37(45.7) | 75(58.1) |  |  |
| ≥8 | 39(48.1) | 36(27.9) |  |  |
| Surgical margin (n, %) |  |  | 20.265 | <0.001* |
| Negative | 19(23.5) | 71(55.0) |  |  |
| Positive | 62(76.5) | 58(45.0) |  |  |
| Perineural invasion (n, %) |  |  | 0.115 | 0.735 |
| Yes | 65(80.2) | 101(78.3) |  |  |
| No | 16(19.8) | 28(21.7) |  |  |
| Lymphovascular invasion (n, %) |  |  | 2.814 | 0.093 |
| Yes | 26(32.1) | 28(21.7) |  |  |
| No | 55(67.9) | 101(78.3) |  |  |
| cT stage (n, %) |  |  | 1.359 | 0.244 |
| T2 | 43(53.1) | 79(61.2) |  |  |
| T3~T4 | 38(46.9) | 50(38.8) |  |  |

*means the p-value<0.05 is considered statistically significant; BMI, body mass index；PSA, prostate specific antigen; PV, prostate volume; PSAD, prostate specific antigen density；BPC, biopsy positive cores; GS, Gleason score, cT stage, clinical T stage.
